# Synthesis Method of Unsolvated Organic Derivatives of Metal Borohydrides

**DOI:** 10.3390/ma15238653

**Published:** 2022-12-05

**Authors:** Wojciech Wegner, Karol J. Fijalkowski

**Affiliations:** Centre of New Technologies, University of Warsaw, ul. Banacha 2c, 02-097 Warsaw, Poland

**Keywords:** synthesis method, borohydrides, organic compounds, organic solvents

## Abstract

A new, scalable, wet-chemistry single-pot method of synthesising pure unsolvated organic derivatives of metal borohydrides is presented. The metathetic reaction in a weakly coordinating solvent is exemplified by the synthesis of [(n-C_4_H_9_)_4_N][Y(BH_4_)_4_] and [Ph_4_P][Y(BH_4_)_4_] systems. For the latter compound, the crystal structure was solved and described. Organic borohydride salts obtained by the new method can find various applications, e.g., can be used as precursors in synthesis of hydrogen-rich mixed-metal borohydrides—promising materials for solid-state chemical storage of hydrogen.

## 1. Introduction

Metal borohydrides and their derivatives comprise extraordinary gravimetric hydrogen content (up to 18.5 wt.% for LiBH_4_) and, therefore, are discussed as potential solid hydrogen storage materials for on-board applications. Organic derivatives of borohydride salts show even larger nominal hydrogen content which increases as a result of the intercalation of organic (i.e., hydrocarbon) groups to the *M*–BH_4_ matrix, which have greater gravimetric hydrogen content than most *M*–BH_4_ salts. The introduction of organic groups to borohydride systems can also enhance the plasticity of these materials, which can result in their improved volumetric packing. Until now, a number of borohydride systems have been reported and various possible applications have been discussed [1,2,3,4].

Classically, metal borohydrides were used as selective reducing agents in chemical synthesis [5,6]. Over the last decades, an increased interest on novel borohydride systems could be attributed mainly to their potential use as hydrogen storage materials; however, various new possible applications of borohydride materials were reported and discussed, i.e., pyrolytic synthesis of metal borides [7,8,9,10] (e.g., superconducting MgB_2_ [11]), pyrolytic synthesis of pure boron nitride [12], laboratory source of diborane [13], polymerisation catalyst (e.g., ε-caprolactone [14]) or direct capture and reduction of CO_2_ (e.g., tetraalkylammonium borohydrides [15]). In addition, many lithium containing borohydride systems exhibit good Li^+^ ion conductivity [16,17,18,19,20]. Recently, magnetic properties of borohydride systems were investigated, as borohydride anion, namely BH_4_^−^, was found to act as a medium of (relatively weak) magnetic superexchange; just as well-known O^2−^ and F^−^ ions [21]. Recently, it was also reported that organic derivatives of lanthanides borohydrides can exhibit room temperature luminescence, together with low temperature field-induced slow magnetic relaxation, placing them as a new family of luminescent single-molecule magnets [22]. What is more, organic derivatives of metal borohydrides can be used as precursors for the synthesis of inorganic mixed-metal borohydrides in a pure form via a wet chemistry approach, suitable for industrial scale synthesis [23,24,25].

Currently, most of the methods of synthesis of specific borohydride salts are using (directly or indirectly) LiBH_4_ or NaBH_4_ as reactants. Sodium borohydride is obtained on an industrial scale using the Bayer process or Brown–Schlesinger process. Lithium borohydride can be obtained in reaction of NaBH_4_ with LiCl or LiBr [26]. A commonly used method of the synthesis of other metal borohydrides and their inorganic derivatives, considers high energy milling (dry mechanochemical synthesis) [2,4,27]. Unfortunately, this approach has severe drawbacks due to the low scale of the mechanochemical method, including possible substitution of BH_4_^−^ anions by chlorides, possible LiCl building in the borohydride structure and so-called ‘dead mass issue’ when by-products cannot be separated from the main product [2,4,7,28].

The above-mentioned drawbacks of dry mechanochemical synthetic methods stimulated the development of solvent-mediated processes, that were expected to ease a scalable synthesis of high purity products. Recently, a method of synthesis of inorganic mixed-metal borohydrides employing well-soluble organic precursors was reported [23,24,25]. It utilises organic metal borohydrides [*Cat*]_x_*M*_y_(BH_4_)_z_ and salts *M*′[*An*] of desired metal cations, where [*Cat*] and [*An*] are weakly coordinating ions, e.g., [*Cat*] = [Bu_4_N]^+^ or [Ph_4_P]^+^; [*An*] = [Al(OC(CF_3_)_3_)_4_]^−^ [29]. The reaction takes place in weakly coordinating solvents with low basicity (e.g., DCM) [30], as exemplified for *M*[Y(BH_4_)_4_] in the state of the art [24]:

*M*[*An*] + [*Cat*][Y(BH_4_)_4_] → *M*[Y(BH_4_)_4_]↓ + [*Cat*][*An*] (1)

where *M* = Li, Na, K, Rb, Cs and the reaction conducted in dichloromethane (DCM). *M*[*An*] for various *M* can be obtained in a pure form with the use of the solvent [29]. Of importance is that this method utilises organic derivatives of metal borohydrides as one of the precursors, which need to be previously obtained in high purity. This was one of the main drawbacks of this method, since such materials are not commercially available and can only be synthesised using the mechanochemical method on a laboratory scale.

The state of the art comprises four mechanochemical methods of synthesising organic derivatives of metal borohydrides, all employing high energy milling. The first method was used for synthesis of [Me_4_N][Y(BH_4_)_4_] and [n-Bu_4_N][Y(BH_4_)_4_] [31,32], where the reaction takes place between two one-cation borohydrides, namely, between Y(BH_4_)_3_ and [*Cat*]BH_4_, [*Cat*] = [Me_4_N] or [n-Bu_4_N] (Equation (2)). The second method was reported for the synthesis of [Me_4_N][Sc(BH_4_)_4_], [n-Bu_4_N][Sc(BH_4_)_4_] and [n-Bu_4_N][*RE*(BH_4_)_4_] (*RE* = Dy, Dy_0.1_Y_0.9,_ Ho, Tm, Yb) [22,33,34], where the reaction takes place between [*Cat*]BH_4_, LiBH_4_ and rare earth chlorides or mixture of chlorides, namely, ScCl_3_ or *RE*Cl_3_ or a mixture of DyCl_3_ with YCl_3_ (Equation (3)). The third method was used for the synthesis of [Ph_4_P][*RE*(BH_4_)_4_] (*RE* = Dy, Dy_0.1_Y_0.9_) [22], where a reaction takes place between [Ph_4_P]Cl, LiBH_4_ and rare earth chlorides (Equation (4)). The fourth method was reported for the synthesis of [Ph_4_P][Sc(BH_4_)_4_] and [Ph_4_P][Zn_2_(BH_4_)_5_] [23,33], where a lithium derivative of scandium or zinc borohydride was reacted with Ph_4_PCl. All those reactions can be generalised to the following reaction Schemes ((2)–(5)), respectively:

x [*Cat*](BH_4_)_m_ + y *M*(BH_4_)_n_ → [*Cat*]_x_*M*_y_(BH_4_)_z_ (2)

a [*Cat*](BH_4_)_m_ + b *MX*_n_ + c *M*′(BH_4_)_o_ → [*Cat*]_x_*M*_y_(BH_4_)_z_ + d *M*′_e_*X*_f_ (3)

a [*Cat*]*Y*_m_ + b *MX*_n_ + c *M*′(BH_4_)_o_ → [*Cat*]_x_*M*_y_(BH_4_)_z_ + d *M*′_e_*X*_f_*Y*_g_ (4)

a *M*′_c_*M*_d_(BH_4_)_md+cn_ + b [*Cat*]*X*_o_ → [*Cat*]_x_*M*_y_(BH_4_)_z_ + e *M*′_f_*X*_g_ (5)

The mechanochemical reactions described above, although they allow successful synthesis of organic derivatives of metal borohydrides, exhibit serious drawbacks. The biggest issue regards the scale of synthesis which, in most cases, does not exceed 1 or 2 mmol of the product (less than 1 g) in one batch, which is suitable for laboratory scale use only. What is more, obtaining a single product in high purity and even distribution of a metal of the same size is not possible solely using mechanochemical treatment and requires the additional step of recrystallisation of the main product, otherwise, a mixture of two or more single-metal organic borohydrides is obtained (e.g., the case of [Bu_4_N][Dy_0.1_Y_0.9_(BH_4_)_4_] [22]). On top of that, high energy milling leads to nano-contamination of the product with particles of the abrasing milling vessel which can hinder their use as, e.g., conducting or magnetic materials. Moreover, mechanochemical reactions frequently lead towards amorphic products needing further treatment to crystallise (e.g., long-time annealing at high temperatures) [35], which can result in decomposition of the material. Incomplete conversion of the reagents into the desired products was also reported [7], which can be interpreted as contamination of the product with the reactants. Further, the mechanochemical approach might not be suitable to synthesise liquid products. For the methods according to Equations (2), (3) and (5), it is also very important that the limiting factor is the availability and possibility of obtaining good quality reactants (e.g., simple borohydrides or complex inorganic borohydrides in pure form) and their reactivity, which may be insufficient for reactions leading to more complex borohydrides.

Most of the above-mentioned drawbacks of the mechanochemical methods apply to the synthesis of organic derivatives of metal borohydrides, [*Cat*]_x_*M*_y_(BH_4_)_z_, which the authors experienced in their laboratory work. Synthesis of [*Cat*]_x_*M*_y_(BH_4_)_z_ using the known methods often leads to partially amorphous products, sometimes in liquid form, often contaminated with unreacted reactants or particles of milling vessel material, which makes the product obtained unsuitable for the desired applications. Limitations of the above-mentioned synthetic methods also result in a relatively small number of organic derivatives of metal borohydrides known to this date.

In this study, we present a novel wet-chemistry method of the synthesis of unsolvated organic derivatives of metal borohydrides, which allows us to freely design the composition of novel organic derivatives of metal borohydrides and to obtain the desired products in a pure form and with good crystallinity in a one step process. The compounds obtained using this novel method can be used as precursors to synthesise hydrogen-rich mixed-metal borohydrides according the Jaroń’s method [23,24,25]. This paper follows a recent patent application (P.442137).

## 2. Materials and Methods

**Reagents handling**. Reagents were handled only inside a glovebox under a dry argon atmosphere (H_2_O < 1 ppm and O_2_ < 1 ppm). Fine quality and high purity anhydrous reagents purchased from Sigma Aldrich were used: YCl_3_ > 99.99%, LiBH_4_ > 95%, (n-C_4_H_9_)_4_N(BH_4_) > 98%, (C_6_H_5_)_4_PCl (Ph_4_PCl) > 98%. Anhydrous dichloromethane (DCM) was used as-purchased from Carl Roth with <30 ppm H_2_O, containing molecular sieves.

**Powder X–Ray diffraction (PXRD)**. PXRD measurements were performed at room temperature using PANalytical X’Pert Pro, with a parallel beam with the CoK_α1_ and CoK_α2_ radiation (intensity ratio of 2:1). Powder samples were sealed under an dry argon atmosphere inside 0.5 mm diameter quartz capillaries.

**Rietveld refinement**. The Jana2006 program [36] was used. For [Ph_4_P][Y(BH_4_)_4_], [Ph_4_P][Dy(BH_4_)_4_] [22] was used as a preliminary structural model. For the background description, 30 Legendre polynomials were used. The peak shape was described by a pseudo-Voigt function. A Berar–Baldinozzi function was used to describe peak asymmetry. To keep reasonable geometries and include hydrogen atoms in the structure’s models, a set of restrains was applied. For all BH_4_^−^ anions, the B–H distances and H–B–H angles were fixed at 1.15 Å and 109.47°, with standard uncertainty, s.u. = 0.001 Å and 0.01°, respectively. For Ph_4_P^+^ cations, the tetrahedral geometry was kept with C–P–C angles fixed at 109.47° with s.u. = 0.01°; planar phenyl rings had all angles fixed at 120° with s.u. = 0.1°, while C–H, C–C and C–P distances were fixed at 1.0 Å, 1.4 Å and 1.8 Å with s.u. = 0.01 Å, 0.1 Å and 0.1 Å, respectively. Hydrogen distances for the three H atoms from each borohydride group coordinating Y^3+^ cations were fixed to be equal with s.u. = 0.01 Å, following the findings for [Ph_4_P][Dy_0.1_Y_0.9_(BH_4_)_4_] [22]. The Atomic Displacement Parameters, ADP, of H atoms were fixed as 1.2 ADP of B atoms.

**CCDC:** [Ph_4_P][Y(BH_4_)_4_]: CCDC number 2217589 contains the supplementary crystallographic data for this paper. These data can be obtained free of charge via http://www.ccdc.cam.ac.uk/conts/retrieving.html or from the Cambridge Crystallographic Data Centre.

**Fourier-transform infrared spectroscopy:** All IR spectra were collected in the 4000–500 cm^−1^ range using a Vertex 80v spectrometer from Bruker. Anhydrous KBr (from Sigma Aldrich, 220 mg per pellet of 12 mm diameter) was used as the pellets’ material.

**Elemental analysis:** C, N and H content was determined using 240 PERKIN ELMER analyser. Cl content was determined by potentiometric argentometric titration with AgNO_3_. Two independent analyses were performed for each type of analysis, with a typical uncertainty of 0.3 wt.%. The figures presented are the mean of the two measured values.

## 3. Results

A novel, room-temperature wet-chemistry approach was designed to synthesise organic derivatives of metal borohydrides in a pure form. The novel synthesis method is based on metathesis—solvent-mediated ion-exchange. In general, the desired organic derivatives of metal borohydrides can be depicted as [*Cat*]_x_*M*_y_(BH_4_)_z_, where [*Cat*] represents an organic cation and *M* represents a metal cation. For the synthesis, [*Cat*]*X*_o_, *MY*_m_ and *M*′(BH_4_)_n_ reactants are used, where *M*′ represents a metal cation not entering the structure of the target compound, while *X* and *Y* represent a monovalent anion (e.g., halide anion) and, in special cases, *X* can represent a BH_4_^−^ anion. The reaction is carried out under an inert atmosphere, according to the following reaction Schemes (6) or (7):

x [*Cat*]*X*_o_ + y *MY*_m_ + w *M*′(BH_4_)_n_ → [*Cat*]_x_*M*_y_(BH_4_)_z_ + a *M*′_b_*X*_c_*Y*_d_ (6)

x [*Cat*](BH_4_)_o_ + y *MY*_m_ + w *M*′(BH_4_)_n_ → [*Cat*]_x_*M*_y_(BH_4_)_z_ + a *M*′_b_*Y*_c_ (7)

where [*Cat*]_x_*M*_y_(BH_4_)_z_ is the main product while *M*′_b_*X*_c_*Y*_d_ or *M*′_b_*Y*_c_ are by-products. Reaction (7) is the special case of the general process described by Reaction (6), where *X* = BH_4_^−^. Reaction is carried out in a solvent which dissolves but does not solvate the main product, [*Cat*]_x_*M*_y_(BH_4_)_z_, while not dissolving the by-products, *M*′_b_*X*_c_*Y*_d_ or *M*′_b_*Y*_c_, e.g., dichloromethane (DCM). The by-product is removed from the reaction mixture as a solid residue by simple filtering. The main unsolvated solid product is obtained by crystallisation.

The novel method was tested upon synthesis of organic yttrium borohydride systems, as they are well known and can act as starting materials in the previously reported method of synthesising mixed metal borohydrides [24] (see Equation (1)); therefore, successful synthesis of these compounds could suggest the first useful application of the method described here. [(n-C_4_H_9_)_4_N][Y(BH_4_)_4_] and [Ph_4_P][Y(BH_4_)_4_] were selected as the target compounds in this study (Table 1). [(n-C_4_H_9_)_4_N][Y(BH_4_)_4_] is a well-known compound synthesised for the first time in 2012 by a mechanochemical method [32], thus, it can act as a good reference to discuss simplicity of the new method and the quality of the product. [Ph_4_P][Y(BH_4_)_4_], however, was not reported earlier with crystallographic data, thus, it can act as an example of the synthesis of novel materials to prove the versatility of the new method.

To synthesise [(n-C_4_H_9_)_4_N][Y(BH_4_)_4_] and [Ph_4_P][Y(BH_4_)_4_], Reactions (8) and (9) were carried out, respectively, scaling on 1 mmol of the main products and using 40 mL of DCM with constant stirring at RT (Table 1). After 12 h, the reactions were quenched, solutions filtered and the solvent evaporated from the clear solutions, giving crystalline products:

[(n-C_4_H_9_)_4_N]BH_4_ + YCl_3_ + 3 LiBH_4_ → [(n-C_4_H_9_)_4_N][Y(BH_4_)_4_] + 3 LiCl (8)

[Ph_4_P]Cl + YCl_3_ + 4 LiBH_4_ → [Ph_4_P][Y(BH_4_)_4_] + 4 LiCl (9)

**Figure 1:**
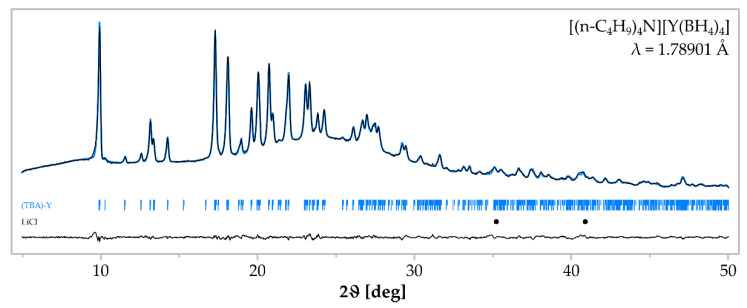
Rietveld analysis of the product sample **1** comprising [(n-C_4_H_9_)_4_N][Y(BH_4_)_4_]. The black profile represents experimental data; blue line—calculated profile. Positions of Bragg reflections for [(n-C_4_H_9_)_4_N][Y(BH_4_)_4_] (blue marks) and LiCl (black dots, not refined) are shown below. A difference curve (between the experimental and calculated profiles) is plotted at the bottom.

**Figure 2:**
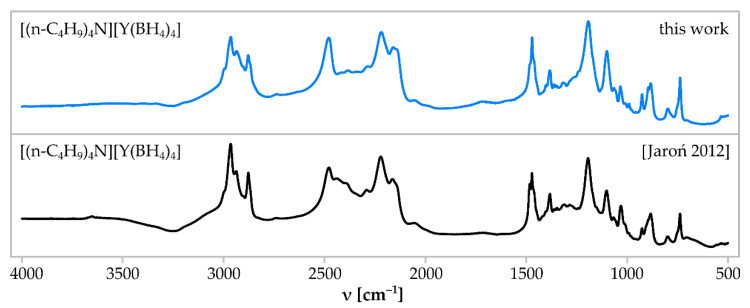
Comparison of IR absorption spectrum of the sample **1** comprising [(n-C_4_H_9_)_4_N][Y(BH_4_)_4_] (**top**, blue line) and a reference spectrum of [(n-C_4_H_9_)_4_N][Y(BH_4_)_4_] obtained in a classical mechanochemical method (**bottom**, black line) [32].

In the reaction (8), a good crystallinity, pure [(n-C_4_H_9_)_4_N][Y(BH_4_)_4_] was obtained as the only detected product in sample **1**. According to Rietveld analysis of the PXRD pattern of sample **1**, [(n-C_4_H_9_)_4_N][Y(BH_4_)_4_] is the only crystalline product (Figure 1) (*P* 2_1_/c; *a* = 11.0453 (5) Å; *b* = 20.0099 (9) Å; *c* = 14.7204 (8) Å; β = 127.98°; V = 2564.44 Å^3^; Z = 4). The IR spectrum of sample **1** (Figure 2) proves the purity of the product, since it is almost identical as a reference spectrum of pure [(n-C_4_H_9_)_4_N][Y(BH_4_)_4_] obtained in a mechanochemical method and purified [32]. The high purity of the obtained [(n-C_4_H_9_)_4_N][Y(BH_4_)_4_] proves that the novel synthetic approach is advantageous over the previously reported mechanochemical method, giving the same product contaminated with residual LiCl and, thus, needing further separation and crystallisation steps to receive the compound in a pure form [32].

**Figure 3:**
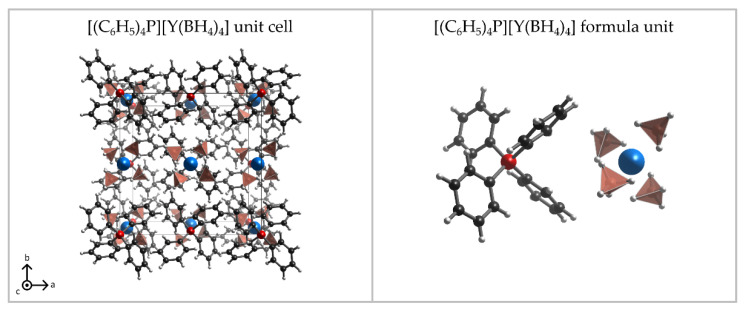
Visualisation of the unit cell (**left**) and the asymmetric unit (**right**) of the crystal structure of [Ph_4_P][Y(BH_4_)_4_]. Atom code—C: black, B: orange, H: white, P: red, Y: blue.

**Figure 4:**
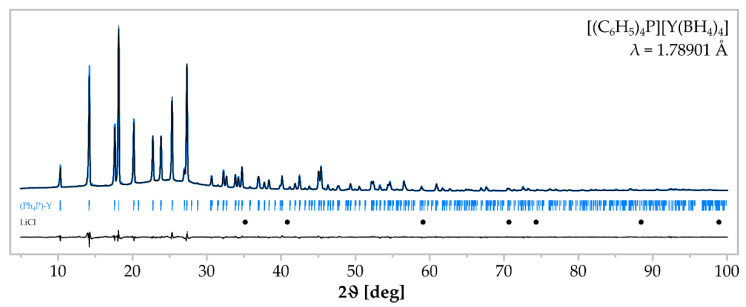
Rietveld analysis of the product sample **2** comprising [Ph_4_P][Y(BH_4_)_4_]. The black profile represents experimental data; blue line—calculated profile. Positions of Bragg reflections for [Ph_4_P][Y(BH_4_)_4_] (blue marks) and LiCl (black dots, not refined) are shown below. A difference curve (between the experimental and calculated profiles) is plotted at the bottom.

**Figure 5:**
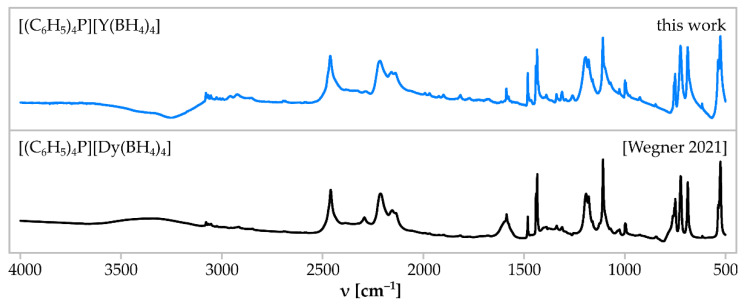
Comparison of IR absorption spectrum of the sample **2** comprising [Ph_4_P][Y(BH_4_)_4_] (top, blue line) and a reference spectrum of [Ph_4_P][Dy(BH_4_)_4_] obtained in a classical mechanochemical method (bottom, black line) [22].

The Reaction (9) gave a crystalline product **2**. The crystal structure of the main product was determined as the expected [Ph_4_P][Y(BH_4_)_4_], which was found to be isostructural to known [Ph_4_P][*RE*(BH_4_)_4_], *RE* = Dy [22], Tm [37] (Figure 3, Table 2). It crystallises in the tetragonal system, *I*4_1_/a space group, comprising alternating [Ph_4_P]^+^ and [Y(BH_4_)_4_]^−^ building blocks, arranged linearly along the *c* axis. Rietveld refinement of the powder pattern of the sample **2** confirms the high purity of the product of Reaction (9) since it does not show signals of the by-product LiCl (Figure 4). The high purity of the sample **2** is also proven by IR spectra, comprising only the bands present in isostructural [Ph_4_P][Dy(BH_4_)_4_] [22] (Figure 5), and the results of elemental analysis, reproducing the theoretical composition of the compound and showing no contamination by LiCl (Table 3).

## 4. Conclusions and Discussion

A novel wet-chemistry single-pot approach towards the synthesis of unsolvated organic derivatives of metal borohydrides was designed and successfully tested upon synthesis of [(n-C_4_H_9_)_4_N][Y(BH_4_)_4_] and [Ph_4_P][Y(BH_4_)_4_]. The compounds received according to the novel method are suitable to act as precursors in Jaroń’s synthesis method of unsolvated hydrogen-rich mixed-metal borohydrides [23,24,25]. The novel procedure considers the reaction between three reactants: an organic salt (halide or borohydride) acting as a source of organic cations, a metal halide acting as a source of desired metal cations and a simple borohydride salt acting as a source of borohydride anions. The reaction is carried out in a weakly solvating solvent (e.g., DCM) for 12 h with stirring, then, the reaction mixture is filtered to separate solid residual by-products to obtain a clear solution of the desired organic derivative of metal borohydride. The final product is received in a pure solid form after crystallisation. Interestingly, only one reactant of the three starting materials is soluble in the solvent (organic salt), while two others (metal halide, metal borohydride) stay insoluble. Two variations in the novel method were tested, one employing an organic borohydride salt as a starting material (sample **1**) and one using an organic chloride salt (sample **2**). In both cases, pure organic derivative of yttrium borohydride was obtained, [*Cat*][Y(BH_4_)_4_], where [*Cat*] = [(n-C_4_H_9_)_4_N] and [Ph_4_P], respectively. While the exact mechanism of this reaction is not known yet, the novel solvent-mediated method of obtaining highly pure organic derivatives of metal borohydrides was proven to be highly effective, one-pot and possible to scale up.

## 5. Patents

The novel synthesis approach presented in this study is also the subject matter of a patent application submitted in the Polish Patent Office on 26 June 2022, no. P.442137 [38].

## Figures and Tables

**Table 1:** Reactions performed using a new method of synthesis of an organic derivative of metal borohydrides: samples names, reactants used, reaction conditions and crystalline products obtained.

Sample	Reactants (mmol)	Reaction Condition	Crystalline Products
**1**	[(n-C_4_H_9_)_4_N]BH_4_, YCl_3_, 3 LiBH_4_	Reaction (8), stirring 12 h in 40 mL DCM, filtration, evaporation of the solvent	[(n-C_4_H_9_)_4_N][Y(BH_4_)_4_]
**2**	[Ph_4_P]Cl, YCl_3_, 4 LiBH_4_	Reaction (9), stirring 12 h in 40 mL DCM, filtration, evaporation of the solvent	[Ph_4_P][Y(BH_4_)_4_]

**Table 2:** Comparison of unit cell parameters for [Ph_4_P][Y(BH_4_)_4_] and isostructural compounds.

Parameter	[Ph_4_P][Y(BH_4_)_4_]	[Ph_4_P][Tm(BH_4_)_4_] [37]	[Ph_4_P][Dy(BH_4_)_4_] [22]
Space group	*I* 4_1_/a	*I* 4_1_/a	*I* 4_1_/a
a (Å)	14.3628 (6)	14.344 (2)	14.373 (2)
c (Å)	13.5567 (7)	13.517 (3)	13.560 (3)
α = β = γ (°)	90	90	90
V (Å^3^)	2796.6 (2)	2781.13	2801.3 (10)
Z	4	4	4

**Table 3:** Comparison of theoretical elemental composition of [Ph_4_P][Y(BH_4_)_4_] with the results of elemental analysis (C, H, N, Cl) of the product sample **2**.

Element	Theoretical Value (%)	Experimental Value (%)
*C*	59.1	58.3
*Y*	18.2	–
*B*	8.9	–
*H*	7.4	7.3
*P*	6.4	–
*N*	–	0
*Cl*	–	0

## Data Availability

Not applicable.
